# Supplementary material for: TGFβ signaling curbs cell fusion and muscle regeneration
Source: Nat Commun. 2021 Feb 2;12:750. doi: 10.1038/s41467-020-20289-8 (PMC7854756; doi:10.1038/s41467-020-20289-8)
Supplement: Supplementary file 1 — Supplementary Information [file 41467_2020_20289_MOESM1_ESM.pdf]

Supplementary information

-

TGFβ signaling curbs cell fusion and muscle regeneration

Girardi et al., 2020

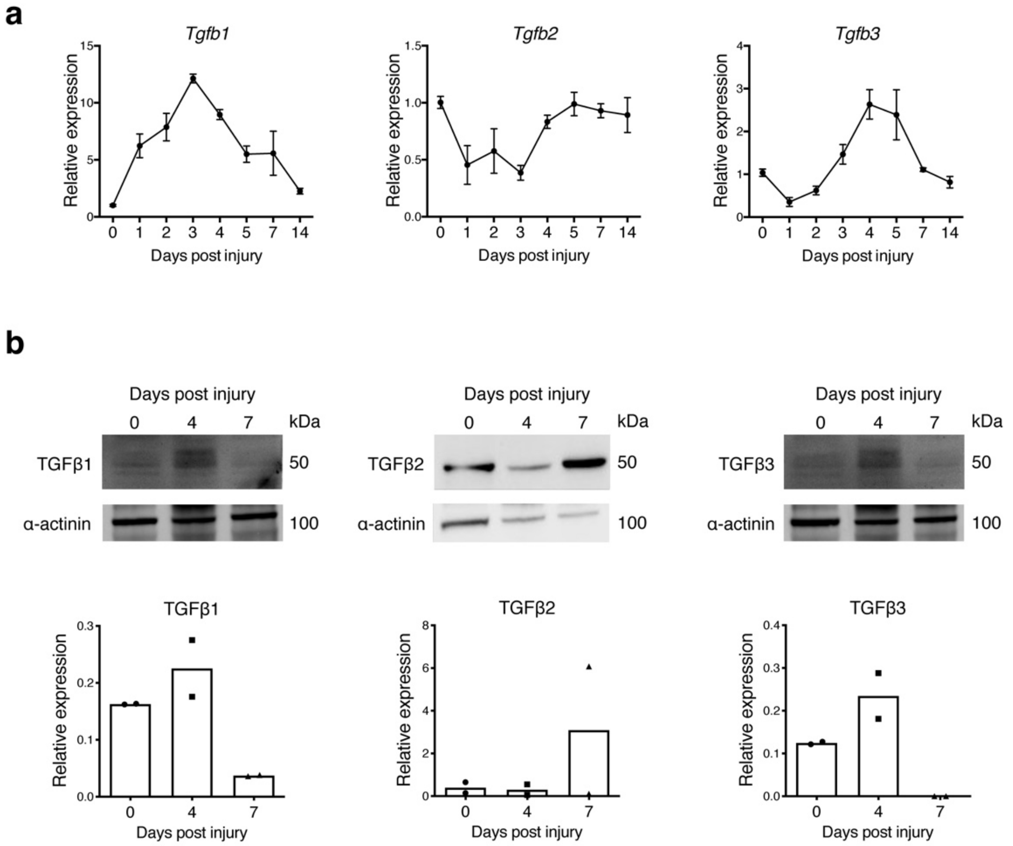

Supplementary Figure 1 | Expression profiles of TGFβ isoforms *in vivo*.
a, qRT-PCR analysis of *Tgfb1*, *Tgfb2* and *Tgfb3* transcripts expression during muscle tissue regeneration induced by CTX injection shows specific expression profiles. N=3 biologically independent experiments. **b,** Western blot analysis of TGFβ1, TGFβ2 and TGFβ3 protein expression. Protein were extracted from 0, 4 and 7 d.p.i. regenerating TA muscles. N=2 biologically independent experiments. Source data are provided as a Source Data file.

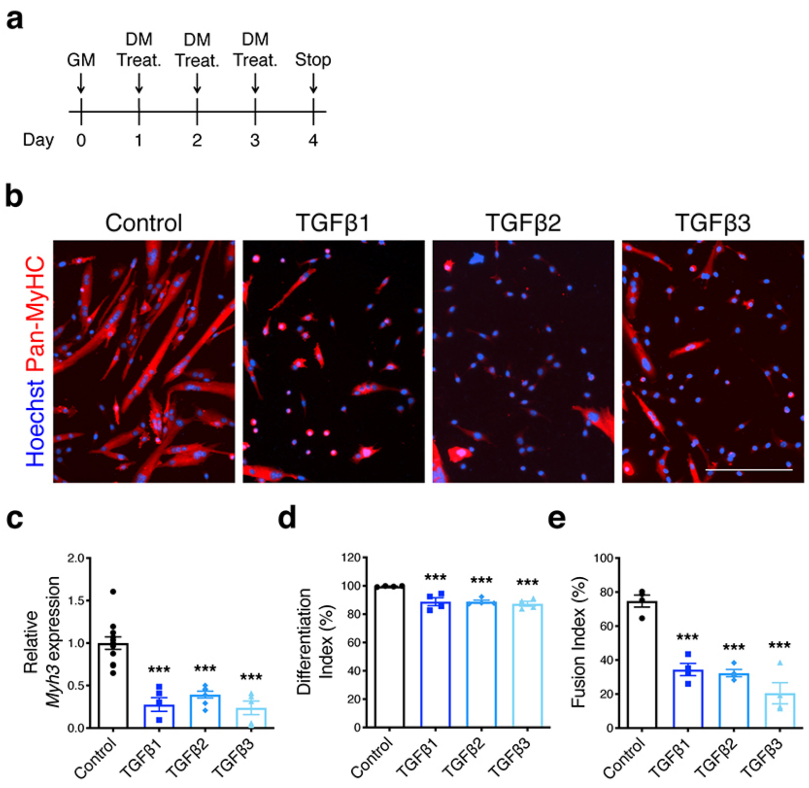

Supplementary Figure 2 | TGFβ signaling effects on *in vitro* myogenic differentiation.
a, Experimental scheme. Primary myoblasts were induced to differentiate in medium containing TGFβ recombinant proteins. **b,** Immunofluorescent staining for Pan-MyHC of 3-days differentiated myotubes. N=4 primary cell cultures. **c,** qRT-PCR analysis for *Myh3* (Embryonic MyHC) transcript expression of 3-days differentiated primary myoblasts indicates that stimulation of the pathway downregulates *Myh3* expression compared to the control. N=12 (Control), 5 (TGFβ1), 6 (TGFβ2), 5 (TGFβ3) biologically independent experiments. **d,** Percentage of Pan-MyHC-expressing cells of 3-days differentiated primary myoblasts. N=4 biologically independent experiments. **e,** Fusion index of 3-days differentiated primary myoblasts shows that TGFβ stimulation inhibits fusion. N=4 biologically independent experiments. Scale bars: **b,** 400µm. Data are presented as mean ± SEM. from at least three independent experiments. ***P<0.001, compared with Control (Unpaired two-tailed Student's t-test). Source data are provided as a Source Data file.

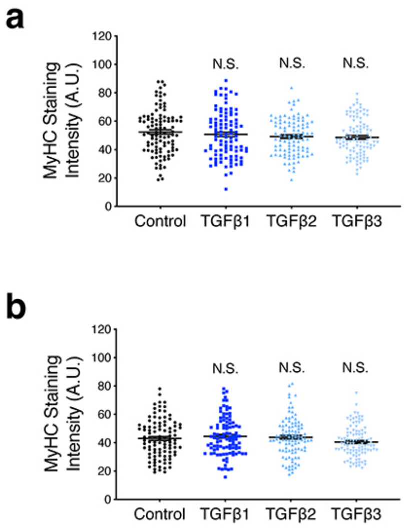

Supplementary Figure 3 | Analysis of myotube maturation in TGFβ-treated cultures.
a, Quantification of MyHC staining intensity of re-plated myotubes (related to Figure 3) reveals that TGFβ activation does not alter myotube maturation levels. **b,** Quantification of MyHC staining intensity of 3-days differentiated myotubes (related to Figure S2) shows that TGFβ stimulation does not impact myotube maturation levels. N=100 cells for each condition examined over 5 biologically independent experiments. Data are presented as mean ± SEM. N.S.=Not significant compared with Control (Unpaired two-tailed Student's t-test). Source data are provided as a Source Data file.

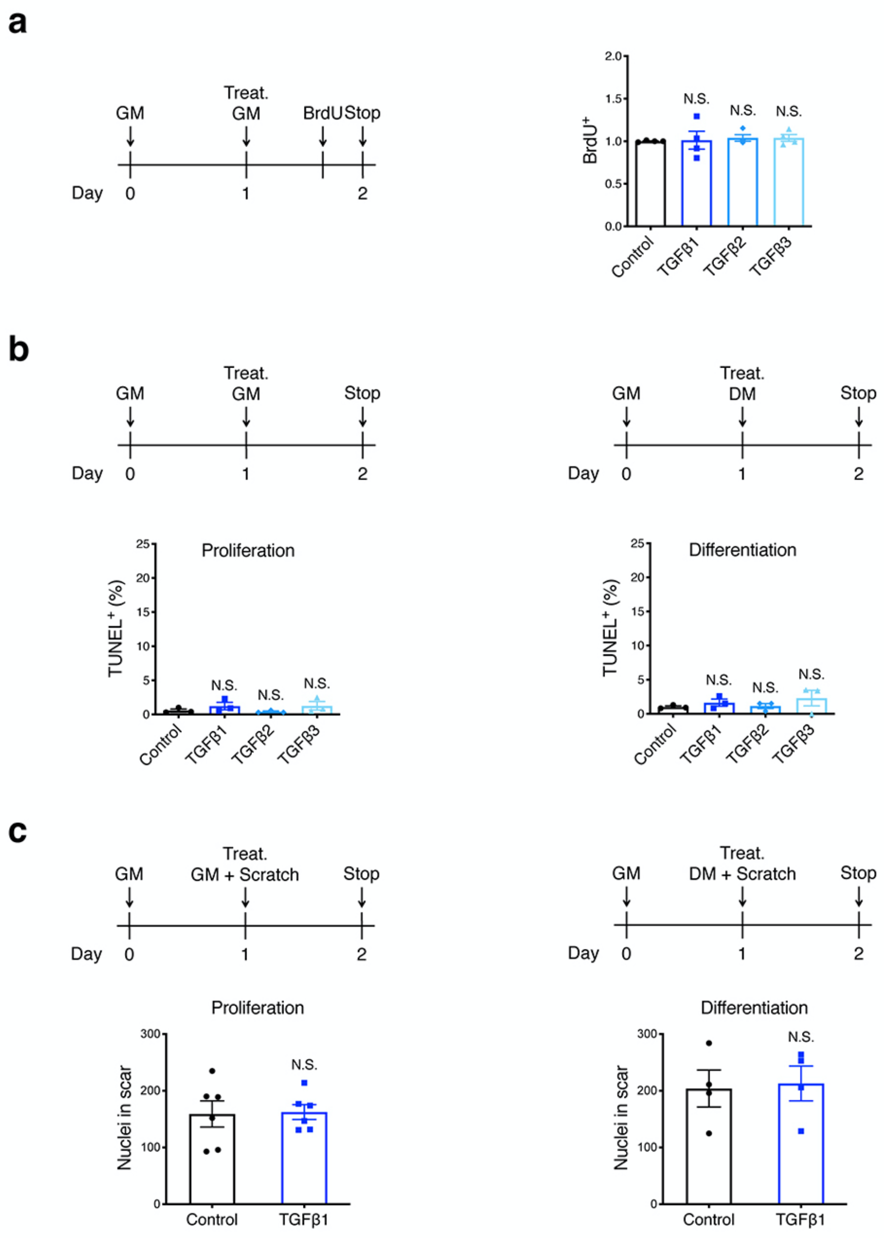

Supplementary Figure 4 | TGFβ signaling does not affect myoblast proliferation, death and motility.

a, Primary myoblasts were treated with TGFβ recombinant proteins for 24h and incubated with BrdU for the last 40 minutes before fixation. Quantification of $BrdU^{+}$ cells shows no differences between treatments. N=4 biologically independent experiments. **b,** Primary myoblasts were treated with TGFβ recombinant proteins for 24h in proliferating or differentiating conditions. $TUNEL^{+}$ cells were quantified, and no particular death rate differences were detected. N=3 biologically independent experiments. **c,** Primary myoblasts were treated with TGFβ1 for 24h in proliferating or differentiating conditions. When treated, cell layer was scratched and washed with PBS. Scratch-wound images were taken after 24 hours of treatment. The quantification of nuclei within the scratch-wound reveals that motility is not affected. N=6 (Proliferation) and 4 (Differentiation) biologically independent experiments. Data are presented as mean ± SEM. N.S.=Not significant, compared with Control (Unpaired two-tailed Student's t-test). Source data are provided as a Source Data file.

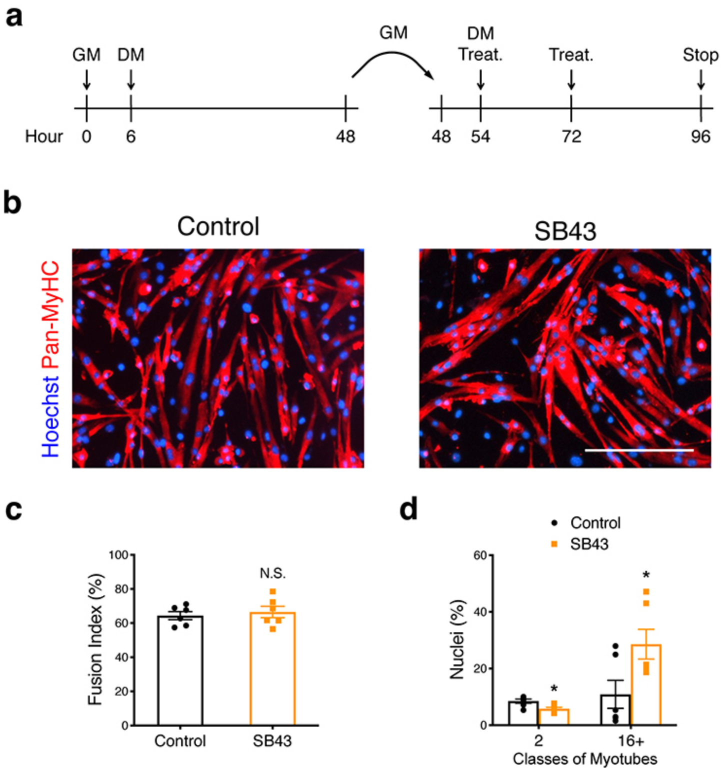

Supplementary Figure 5 | TGFBR1 inhibition increases fusion.
a, Experimental scheme. Primary myoblasts seeded at low density (5000 cells/cm^2) were differentiated for two days, split and re-plated at high density (75000 cells/cm^2) and cultured for two more days. **b,** Immunofluorescent staining for Pan-MyHC of re-plated myocytes cultured for 48 hours. N=6 primary cell lines. **c,** Fusion index of re-plated myocytes reveals no significant differences between control and SB43 conditions. N=6 biologically independent experiments. **d,** Percentage of nuclei in the smallest and largest myotube classes. As compared to the control, SB43 treatment reduces the number of nuclei within small myotubes and strongly increases the percentage of nuclei in the large myotubes. N=6 biologically independent experiments. Scale bars: **b,** 200µm. Data are presented as mean ± SEM. *P<0.05, N.S.=Not significant, compared with Control (Unpaired two-tailed Student's t-test). Source data are provided as a Source Data file.

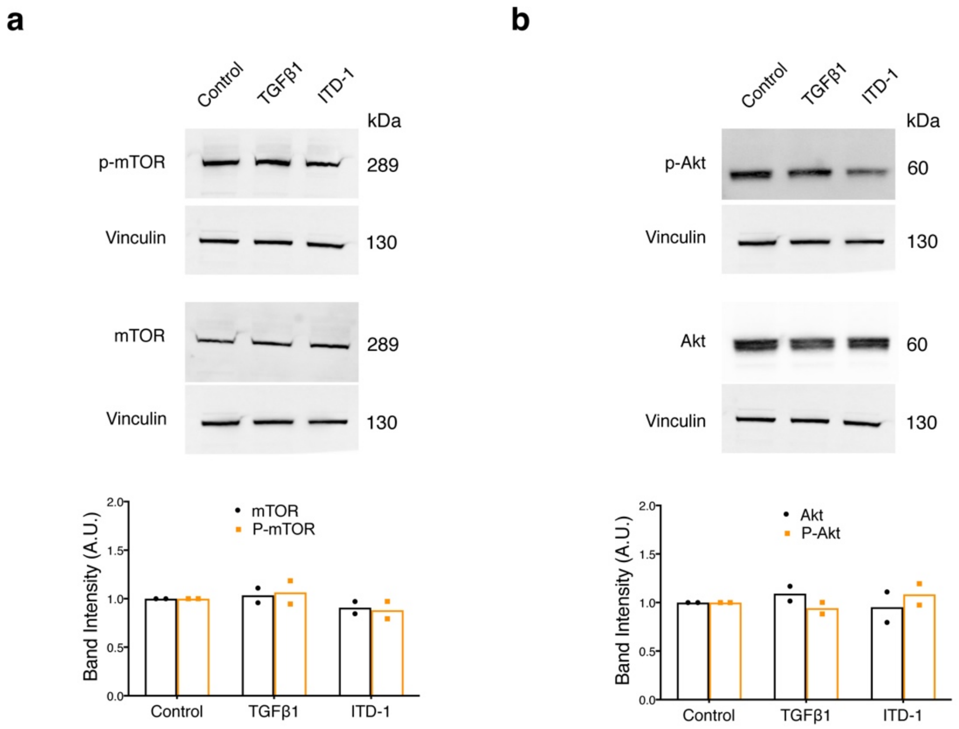

Supplementary Figure 6 | TGFβ signaling does not impact Akt/mTOR pathway.
a, p-mTOR and mTOR western blot analysis of 1-day differentiated myocytes treated with TGFβ1 protein or ITD-1 compound. No significant changes have been observed. **b,** p-AKT and AKT western blot analysis of 1-day differentiated myocytes treated with TGFβ1 proteins or ITD-1 compound. No significant changes have been observed. N=2 biologically independent experiments. Data are presented as mean ± SEM. Of note, VINCULIN blots in **a** and **b** are duplicate images, since from the same membrane, but twice presented for reference purposes. Source data are provided as a Source Data file.

a

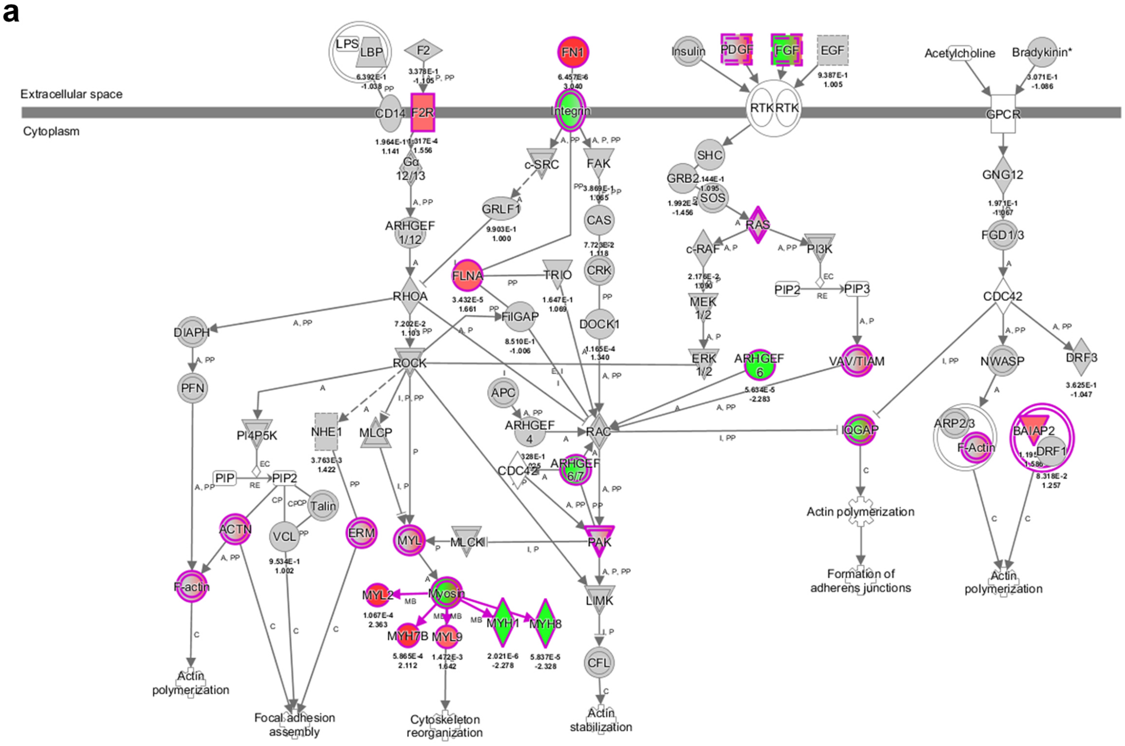

b

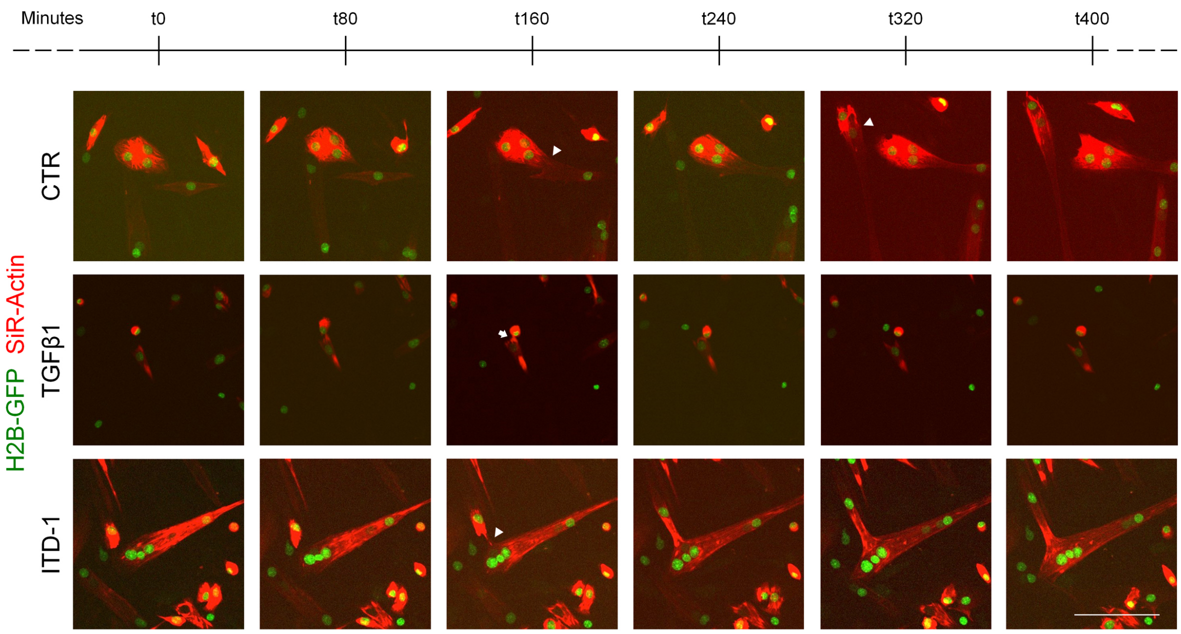

Supplementary Figure 7 | Effects of TGFβ cascade on Actin signaling pathway.
a, Representative scheme of the Actin signaling pathway genes modulated by TGFβ cascade. **b,** Live-imaging frames of pre-differentiated myocytes expressing H2B-GFP and stained with SiR-Actin. Arrowheads indicate fusion events, arrow depicts cell-cell interaction. In control condition, fusion occurs linearly, while ITD-1 treatment allows perpendicular fusion. On the other hand, TGFβ1 allows cell-cell interactions, but blocks fusion. n=6 primary cultures. Scale bars: **b,** 200µm. Source data are provided as a Source Data file.

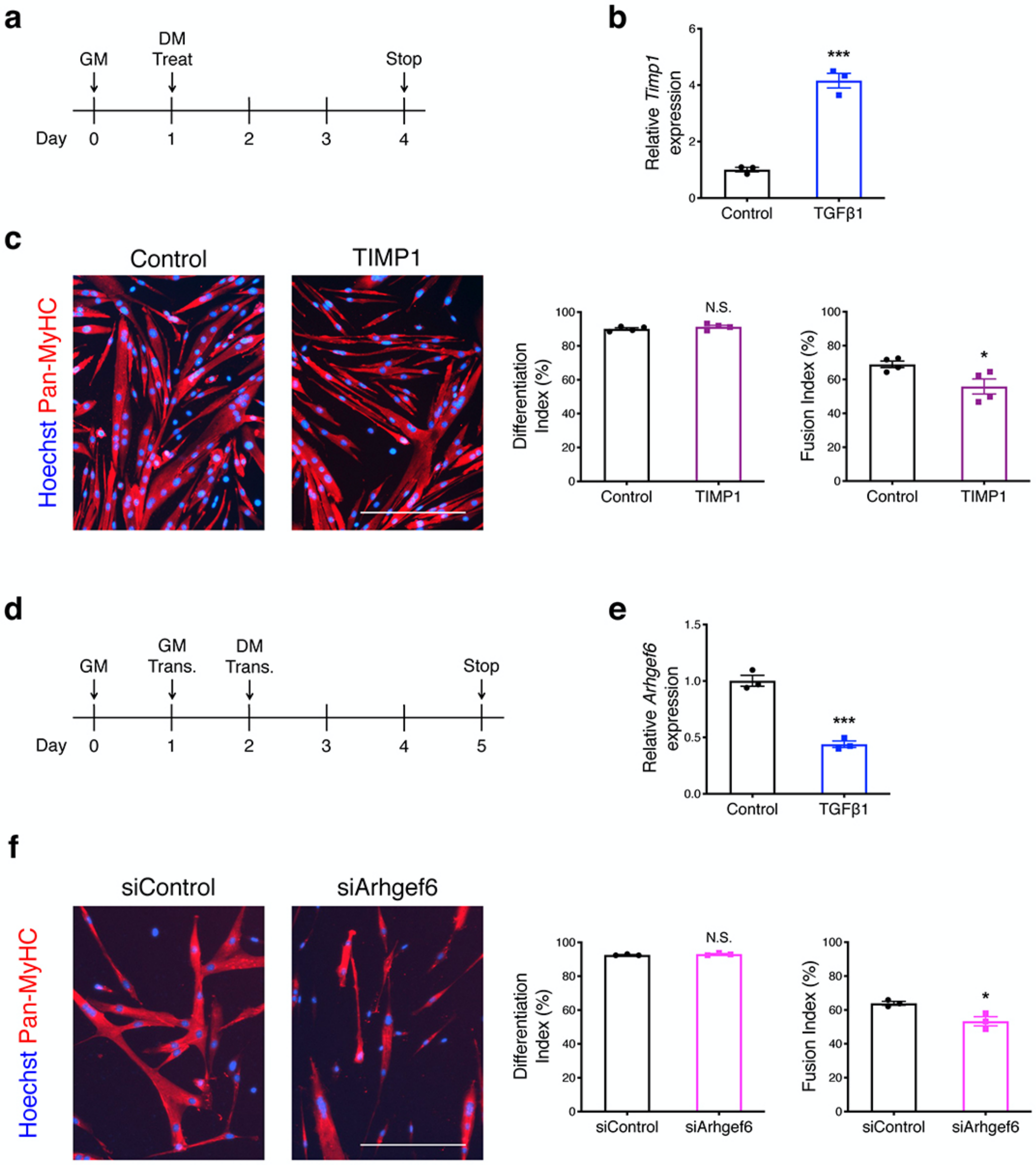

Supplementary Figure 8 | TGFβ-driven effect on fusion is mediated, in part, by Timp1 and Arhgef6.

a, Experimental scheme. Primary myoblasts were induced to differentiate in medium containing TIMP1 recombinant proteins. **b,** *Timp1* expression from transcriptomic analysis on differentiated myocytes treated with either TGFβ1. N=10 biologically independent experiments. **c,** Immunofluorescent staining for Pan-MyHC of 3-days differentiated myotubes. Although differentiation index is not affected by the treatment, fusion index of 3-days differentiated primary myoblasts shows that TIMP1 administration decreases fusion index. N=3 primary cultures. **d,** Experimental scheme. Primary myoblasts were seeded in antibiotic-free growth medium and transfected twice (before and at the onset of differentiation) with siRNA against Arhgef6. **e,** *Arhgef6* expression from transcriptomic analysis on differentiated myocytes treated with TGFβ1. N=10 biologically independent experiments. **f,** Immunofluorescent staining for Pan-MyHC of 3-days differentiated myotubes. Fusion index of 3-days differentiated primary myoblasts shows that Arhgef6 silencing decreases fusion index but does not affect differentiation index. N=3 primary cultures. Scale bars: **b, f,** 200µm. Data are presented as mean ± SEM. *P<0.05, ***P<0.001 N.S.=Not significant, compared with Control (Unpaired two-tailed Student's t-test). Source data are provided as a Source Data file.

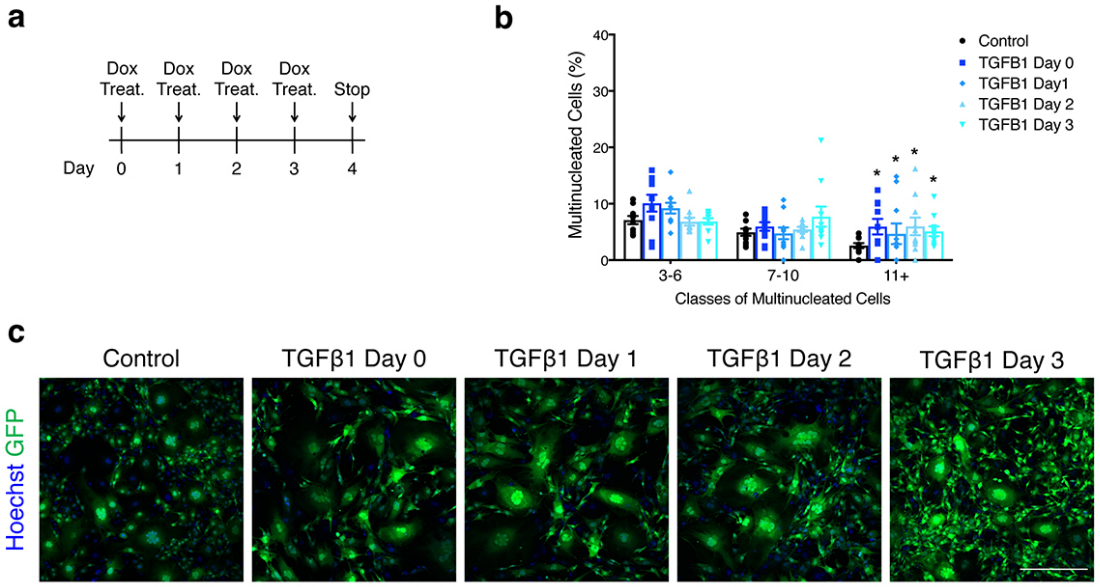

Supplementary Figure 9 | TGFβ signaling exerts its effect independently from Myomaker and Myomerger.

a, Experimental scheme. Dox-Inducible Myomaker- and Myomerger-expressing fibroblasts were used to test TGFβ1 effect on cell-cell fusion. Dox was administrated at day 0 and refreshed every day, while TGFβ1 either at day 0, 1, 2 or 3. **b,** Aggregation index of 4-days Myomaker and Myomerger-expressing fibroblasts showing no significant reduction of the fusion process when TGFβ1 is administrated compared to the control. N=3 biologically independent experiments. **c,** GFP-myomaker-infected fibroblasts, transduced with dox-inducible Myomerger, were visualized with fluorescent microscopy. TGFβ stimulation does not reduce the fusion process. Scale bars: **c,** 400µm. Data are presented as mean ± SEM. **P*<0.05 compared with Control (Unpaired two-tailed Student's t-test). Source data are provided as a Source Data file.

Primer	Sequence
Tbp_Fwd	CCCCACAACTCTTCCATTCT
Tbp_Rev	GCAGGAGTGATAGGGGTCAT
Smad7_Fwd	GGCCGGATCTCAGGCATTC
Smad7_Rev	TTGGGTATCTGGAGTAAGGAGG
Klf10_Fwd	GTGACCGTCGGTTTATGAGGA
Klf10_Rev	AGCTTCTTGGTCGATAGGTGG
Myh3_Fwd	AAGGCCAAAAAGGCCATC
Myh3_Rev	TCTTCTGCTCCCCTTCCA
Tgfb1_Fwd	CTCCCGTGGCTTCTAGTGC
Tgfb1_Rev	GCCTTAGTTTGGACAGGATCTG
Tgfb2_Fwd	ATCGTCCGCTTTGATGTCTC
Tgfb2_Rev	GCTGGGTGGGAGATGTTAAG
Tgfb3_Fwd	AGGATCACCACAACCCACAC
Tgfb3_Rev	ATAAAGGGGGCGTACACAGC
Alk5_Fwd	TTATGAGAGAATGCTGGTATG
Alk5_Rev	AAGAGAGCAGAGTTCCCACGG
Tgfbr2_Fwd	CGGATGTGGAAATGGAAGCC
Tgfbr2_Rev	TGTCGCAAGTGGACAGTCTC
Myogenin_Fwd	GCAATGCACTGGAGTTCG
Myogenin_Rev	ACGATGGACGTAAGGGAGTG

Supplementary Table 1: qRT-PCR primers used in this study

Antibody target	Dilution	Supplier	Reference
Myogenin	1:100	Santa Cruz Biotech.	Sc-52903
Pan-MyHC	1:10	DSHB	MF-20
Laminin	1:400	Abcam	ab11575
Dystrophin	1:200	Thermo Fisher Scie.	RB-9024
Pax7	1:20	DSHB	Pax7
BrdU	1:100	Abcam	Ab6326
Vinculin	1:1000	Abcam	Ab18058
Histone3	1:1000	Cell Signaling Tech.	4499
SMAD2/3	1:5000	Cell Signaling Tech.	8685
p-SMAD2/3	1:5000 (WB) 1:200 (IF)	Cell Signaling Tech.	8828
p-SMAD3	1:200	Abcam	Ab52903
Akt	1:1000	Cell Signaling Tech.	4691
p-Akt	1:1000	Cell Signaling Tech.	4058
Myod1	1:100	Santa Cruz Biotech.	Sc-377460
mTOR	1:1000	Cell Signaling Tech.	2972
p-mTOR	1:1000	Cell Signaling Tech.	2971
TGFβ1	1:500	R&D System	MAB240
TGFβ2	1:500	Abcam	Ab36495
TGFβ3	1:500	R&D System	MAB243
α-Actinin	1:1000	Sigma-Aldrich	A7811

Supplementary Table 2: Antibodies used in this study
