# Supplementary material for: TGFβ signaling curbs cell fusion and muscle regeneration
Source: Nat Commun. 2021 Feb 2;12:750. doi: 10.1038/s41467-020-20289-8 (PMC7854756; doi:10.1038/s41467-020-20289-8)
Supplement: Supplementary file 9 — Reporting Summary [file 41467_2020_20289_MOESM9_ESM.pdf]

nature research

Corresponding author(s): Dr. Fabien Le Grand

Last updated by author(s): Sep 20, 2020

Reporting Summary

Nature Research wishes to improve the reproducibility of the work that we publish. This form provides structure for consistency and transparency in reporting. For further information on Nature Research policies, see Authors & Referees and the Editorial Policy Checklist .

Statistics

For all statistical analyses, confirm that the following items are present in the figure legend, table legend, main text, or Methods section.

n/a	Confirmed	
☐	☒	The exact sample size (n) for each experimental group/condition, given as a discrete number and unit of measurement
☐	☒	A statement on whether measurements were taken from distinct samples or whether the same sample was measured repeatedly
☐	☒	The statistical test(s) used AND whether they are one- or two-sided<br>*Only common tests should be described solely by name; describe more complex techniques in the Methods section.*
☒	☐	A description of all covariates tested
☒	☐	A description of any assumptions or corrections, such as tests of normality and adjustment for multiple comparisons
☐	☒	A full description of the statistical parameters including central tendency (e.g. means) or other basic estimates (e.g. regression coefficient) AND variation (e.g. standard deviation) or associated estimates of uncertainty (e.g. confidence intervals)
☐	☒	For null hypothesis testing, the test statistic (e.g. F, t, r) with confidence intervals, effect sizes, degrees of freedom and P value noted<br>*Give P values as exact values whenever suitable.*
☒	☐	For Bayesian analysis, information on the choice of priors and Markov chain Monte Carlo settings
☒	☐	For hierarchical and complex designs, identification of the appropriate level for tests and full reporting of outcomes
☒	☐	Estimates of effect sizes (e.g. Cohen's d, Pearson's r), indicating how they were calculated

Our web collection on statistics for biologists contains articles on many of the points above.

Software and code

Policy information about availability of computer code

Data collection	For Spinning Disk Imaging, we used MetaMorph® version 7. For hMMT imaging, we used FV10-ASW version 4.2. For RT-qPCR, we used LightCycler480 software version 1.5. Microarray data were analyzed with Partek® Genomic Suite® version 6 and gene networks were identified using the Ingenuity Pathways Analysis® (IPA) version 8.
Data analysis	GraphPad® Prism 6 version 6.0c. Microsoft Excel® version 16.41. ImageJ® version 1.51.

For manuscripts utilizing custom algorithms or software that are central to the research but not yet described in published literature, software must be made available to editors/reviewers. We strongly encourage code deposition in a community repository (e.g. GitHub). See the Nature Research guidelines for submitting code & software for further information.

Data

Policy information about availability of data

All manuscripts must include a data availability statement. This statement should provide the following information, where applicable:

- Accession codes, unique identifiers, or web links for publicly available datasets
- A list of figures that have associated raw data
- A description of any restrictions on data availability

Source data are provided with this paper. Microarray data have been deposited in the NCBI Gene Expression Omnibus database and are accessible through the accession number GSE123425.

Field-specific reporting

Please select the one below that is the best fit for your research. If you are not sure, read the appropriate sections before making your selection.

Life sciences study design

All studies must disclose on these points even when the disclosure is negative.

Sample size	Sample sizes were based on previous experience in the lab (Le Grand, J. Cell Biol, 2012; Rudolf, Cell Reports, 2016). Sample size was determined to be adequate based on the magnitude and consistency of measurable differences between groups.
Data exclusions	No results have been excluded.
Replication	At least three different biological replicates has been performed for each experiment. Most of biological replicates have been performed with two or more technical replicas. Conclusion were made only when all the biological replicates were consistent. All attempts at replication were succesful.
Randomization	Treatment groups were randomly chozen among mice shipped from Janvier Laboratories to our facility.
Blinding	Due to technical/researcher limitations and obvious phenotypes, blinding was not performed for most experiments.

Reporting for specific materials, systems and methods

We require information from authors about some types of materials, experimental systems and methods used in many studies. Here, indicate whether each material, system or method listed is relevant to your study. If you are not sure if a list item applies to your research, read the appropriate section before selecting a response.

Materials & experimental systems

n/a	Involved in the study	
☐	☒	Antibodies
☐	☒	Eukaryotic cell lines
☒	☐	Palaeontology
☐	☒	Animals and other organisms
☒	☐	Human research participants
☒	☐	Clinical data

Methods

n/a	Involved in the study	
☒	☐	ChIP-seq
☒	☐	Flow cytometry
☒	☐	MRI-based neuroimaging

Antibodies

Antibodies used	Myogenin (1:100) Santa Cruz Biotech. Sc-52903; Pan-MyHC (1:10) DSHB MF-20. Laminin (1:400) Abcam ab11575. Dystrophin (1:200) Thermo Fisher Scie. RB-9024. Pax7 (1:20) DSHB Pax7. BrdU (1:100) Abcam Ab6326. Vinculin (1:1000) Abcam Ab18058. Histone3 (1:1000) Cell Signaling Tech. 4499. SMAD2/3 (1:5000) Cell Signaling Tech. 8685. Phospho-SMAD2/3 (1:5000 for WB) (1:200 for IF) Cell Signaling Tech. 8828. Phospho-SMAD3 (1:200) Abcam Ab52903. Akt (1:1000) Cell Signaling Tech. 4691. Phospho-Akt (1:1000) Cell Signaling Tech. 4058. mTOR (1:1000) Cell Signaling Tech. 2972. Phospho-mTOR (1:1000) Cell Signaling Tech. 2971. TGFb1 (1:500) R&D System MAB240. TGFb2 (1:500) Abcam AB36495. TGFb3 (1:500) R&D System MAB243. alpha-Actinin (1:1000) Sigma-Aldrich A7811. Myod1 (1:100) Santa Cruz Biotech. Sc-377460. Alexa488 Goat anti-mouse IgG1, Invitrogen #A-21121. Alexa546 Goat anti-mouse IgG2b, Invitrogen #A-21143. Alexa647 Goat anti-rabbit IgG, Invitrogen #A-21244. HRP goat anti-mouse (1:10000), Jackson Immuno 115-035-003. HRP goat antirabbit (1:10000), Jackson Immuno 111-035-003. Lot number are not provided since the project spanned a four-years period in which different lots were used without any relevant variation.
Validation	Laminin; https://www.abcam.com/laminin-antibody-ab11575.html. Dystrophin; https://www.thermofisher.com/order/catalog/product/RB-9024-P#/RB-9024-P. Pax7; https://dshb.biology.uiowa.edu/PAX7. BrdU; https://www.abcam.com/brdu-antibody-bu175-icr1-proliferation-marker-ab6326.html. Vinculin (discontinued). Histone3; https://www.cellsignal.com/products/primary-antibodies/histone-h3-d1h2-xp-rabbit-mab/4499?Ntk=Products&Ntt=4499. SMAD2/3; https://www.cellsignal.com/products/primary-antibodies/smad2-3-d7g7-xp-rabbit-mab/8685?Ntk=Products&Ntt=8685. Phospho-SMAD2/3; https://www.cellsignal.com/products/primary-antibodies/phospho-smad2-ser465-467-smad3-ser423-425-d27f4-rabbit-mab/8828?Ntk=Products&Ntt=8828. Phospho-SMAD3; https://www.abcam.com/smad3-phospho-s423--s425-antibody-ep823y-ab52903.html. Akt; https://www.cellsignal.com/products/primary-antibodies/akt-pan-c67e7-rabbit-mab/4691?Ntk=Products&Ntt=4691. Phospho-Akt; https://www.cellsignal.com/products/primary-antibodies/phospho-akt-ser473-193h12-rabbit-mab/4058?Ntk=Products&Ntt=4058. mTOR; https://www.cellsignal.com/products/primary-antibodies/mtor-antibody/2972?Ntk=Products&site-search-type=Products&N=4294956287&Ntt=+2972&fromPage=plp&_requestid=1521977. Phospho-mTOR; https://www.cellsignal.com/products/primary-antibodies/phospho-mtor-ser2448-antibody/2971?Ntk=Products&Ntt=2971. TGFb1; https://www.rndsystems.com/products/tgf-beta1-antibody-9016_mab240. TGFb2 https://www.abcam.com/tgf-beta-2-antibody-ab36495.html. TGFb3; https://www.rndsystems.com/products/tgf-beta3-antibody-20724_mab243. alpha-Actinin; https://www.sigmaaldrich.com/catalog/product/sigma/a7811?lang=en&region=US&gclid=CjwKCAjw5Kv7BRBSEiwAXGDElV99uzy7Nr5evsmyylYE6rBkhLGCVM1kKg48RhMQdYAKPlUJxCcM5xoCalEQAvD_BwE. Myod1 ; https://www.scbt.com/p/myod-antibody-g-1?requestFrom=search. Alexa488 Goat anti-mouse IgG1; https://www.thermofisher.com/antibody/product/Goat-anti-Mouse-IgG1-Cross-Adsorbed-Secondary-Antibody-Polyclonal/A-21121. Alexa647 Goat anti-rabbit IgG; https://www.thermofisher.com/antibody/product/Goat-anti-Rabbit-IgG-H-L-Cross-Adsorbed-Secondary-Antibody-Polyclonal/A-21244. Alexa546 Goat anti-mouse IgG2b; https://www.thermofisher.com/antibody/product/Goat-anti-Mouse-IgG2b-Cross-Adsorbed-Secondary-Antibody-Polyclonal/A-21143. HRP goat anti-mouse; https://

www.jacksonimmuno.com/catalog/products/115-035-003. HRP goat anti-rabbit; https://www.jacksonimmuno.com/catalog/products/111-035-003.

Eukaryotic cell lines

Policy information about cell lines

Cell line source(s)	Skeletal muscle-derived primary myoblasts were isolated from wild-type mice using the Satellite Cell Isolation Kit MACS protocol (Miltenyi Biotec).<br>The 3D hMMTs were generated using human immortalized myoblast lines obtained from V. Mouly (AB1167 from fascia lata muscle of a healthy 20-year old male, AB1190 from paravertebral muscle of a healthy 16-year old male, and KM155 from thigh muscle of a healthy 25-year old male) (Mamchaoui et al., 2011).<br>Myomaker and Myomerger-expressing 10T1/2 fibroblasts were obtained as described previously in Leikina et al., 2018.
Authentication	Cell lines were not authenticated.
Mycoplasma contamination	Mycoplasma contamination has never been tested since no aberrant behavior were detected.
Commonly misidentified lines (See ICLAC register)	No cell lines used are listed in the database of commonly misidentified cell lines.

Animals and other organisms

Policy information about studies involving animals; ARRIVE guidelines recommended for reporting animal research

Laboratory animals	Mice of either sex ranging from 3 to 5 months of C57/BL6/N strain were used and purchased from Janvier Laboratories. Animals were exposed to a standard 12:12 light/dark cycles with normal activity and chow, 55% humidity (range 45-65) at 22°C temperature (range 20-24).
Wild animals	No wild animals were used in the study.
Field-collected samples	No field collected samples were used in the study.
Ethics oversight	Experiments were performed at the Centre d'Expérimentation Fonctionnelle (UMS28) Animal Facility following the European regulations for animal care and handling. Experimental animal protocols were performed in accordance with the guidelines of the French Veterinary Department and approved by the Sorbonne Université Ethical Committee for Animal Experimentation.

Note that full information on the approval of the study protocol must also be provided in the manuscript.
