# Supplementary material for: TGFβ signaling curbs cell fusion and muscle regeneration
Source: Nat Commun. 2021 Feb 2;12:750. doi: 10.1038/s41467-020-20289-8 (PMC7854756; doi:10.1038/s41467-020-20289-8)
Supplement: Supplementary file 10 — Description of Additional Supplementary Files [file 41467_2020_20289_MOESM10_ESM.pdf]

Title: Supplementary Movie 1 | Brightfield movie of untreated myoblast during early differentiation.
Description: Experimental scheme: primary myoblasts were seeded at 10000 cells/cm^2 and induced to differentiate. After 12 hours, cells were recorded live for 12 hours. During this time lapse, primary myoblasts are motile, perform multiple cell-cell contacts and start to differentiate. n=6 biologically independent primary cell lines. Scale bar: 400µm.

Title: Supplementary Movie 2 | Brightfield movie during early differentiation of myoblast treated with TGFβ1 recombinant protein.
Description: Experimental scheme: primary myoblasts were seeded at 10000 cells/cm^2 and induced to differentiate with TGFβ1 recombinant protein. After 12 hours, cells were recorded live for 12 hours. The administration of TGFβ1 recombinant protein does not impair myoblast motility and cell-cell contact frequency. n=6 biologically independent primary cell lines. Scale bar: 400µm.

Title: Supplementary Movie 3 | Brightfield movie of untreated myoblast during late differentiation.
Description: Experimental scheme: primary myoblasts were seeded at 10000 cells/cm^2 and induced to differentiate. After 36 hours, cells were recorded live for 24 hours. During late differentiation, differentiated primary myoblasts contact each other and eventually fuse together leading to myotube formation. n=6 biologically independent primary cell lines. Scale bar: 400µm.

Title: Supplementary Movie 4 | Brightfield movie during late differentiation of myoblast treated with TGFβ1 recombinant protein.
Description: Experimental scheme: primary myoblasts were seeded at 10000 cells/cm^2 and induced to differentiate with TGFβ1 recombinant protein. After 36 hours, cells were recorded live for 24 hours. Although TGFβ1 stimulation does not alter cell-cell contact frequency, the majority of contacting myoblasts fail to fuse and to form syncytia. n=6 biologically independent primary cell lines. Scale bar: 400µm.

Title: Supplementary Movie 5 | Live imaging movie of co-cultured pre-differentiated untreated myoblasts.
Description: Experimental scheme: H2B-GFP primary myoblasts were seeded at low density (5000 cells per cm^2), treated with TGFβ1 protein or ITD-1 compound, stained with SiR-Actin and differentiated for 2 days. Membrane-tdTomato primary myoblasts were seeded at low density (5000 cells per cm2) and were differentiated for two days. Both populations were split and co-cultured (50/50) at high density (75000 cells per cm^2) and cultured for two more days. In the last 40 hours, cells were recorded live by confocal microscopy. The two primary cultures are able to fuse together and lead to the formation of heterologous myotubes (double positive for SiR-Actin and tdTOMATO). n=6 biologically independent primary cell lines. Scale bar: 200µm.

Title: Supplementary Movie 6 | Live imaging movie of co-cultured pre-differentiated myoblasts treated or not with TGFβ1 protein.
Description: Experimental scheme: H2B-GFP primary myoblasts were seeded at low density (5000 cells per cm^2), treated with TGFβ1 protein or ITD-1 compound, stained with SiR-Actin and differentiated for 2 days. Membrane-tdTomato primary myoblasts were seeded at low density (5000 cells per cm2) and were differentiated for two days. Both populations were split and co-cultured (50/50) at high density (75000 cells per cm^2) and cultured for two more days. In the last 40 hours, cells were recorded live by confocal microscopy. Prior TGFβ1 signaling stimulation of H2B-GFP primary myoblasts reduces heterologous fusion between the 2 populations. n=6 biologically independent primary cell lines. Scale bar: 200µm.

Title: Supplementary Movie 7 | Live imaging video of co-cultured pre-differentiated myoblasts treated or not with ITD-1 compound.
Description: Experimental scheme: H2B-GFP primary myoblasts were seeded at low density (5000 cells per cm^2), treated with TGFβ1 protein or ITD-1 compound, stained with SiR-Actin and differentiated for 2 days. Membrane-tdTomato primary myoblasts were seeded at low density (5000 cells per cm2) and were differentiated for two days. Both populations were split and co-cultured (50/50) at high density (75000 cells per cm^2) and cultured for two more days. In the last 40 hours, cells were recorded live by confocal microscopy. Prior treatment of ITD-1 compound of H2B-GFP primary myoblasts leads to the formation of large syncytia and increases heterologous fusion events. n=6 biologically independent primary cell lines. Scale bar: 200µm.

Title: Supplementary Data 1 | Changes in gene expression following modulation of TGFβ signaling pathway in primary myocytes.
Description: Experimental scheme: primary myoblasts were differentiated for 24hours and resulting myocytes were treated with TGFβ1 protein or ITD-1 compound. After 24hours, RNA was extracted from cell cultures and

Gene expression was analyzed using Affymetrix Clariom[tm] microarrays. n=3 biologically independent primary cell lines.
